# Allowing for non-adherence to treatment in a randomized controlled trial of two antidepressants (citalopram *versus* reboxetine): an example from the GENPOD trial

**DOI:** 10.1017/S0033291714000221

**Published:** 2014-03-03

**Authors:** N. J. Wiles, K. Fischer, P. Cowen, D. Nutt, T. J. Peters, G. Lewis, I. R. White

**Affiliations:** 1 School of Social and Community Medicine, University of Bristol, UK; 2 Estonian Genome Centre, University of Tartu, Estonia; 3 Department of Psychiatry, University of Oxford, UK; 4 Department of Neuropsychopharmacology, Imperial College London, UK; 5 School of Clinical Sciences, University of Bristol, UK; 6 Mental Health Sciences Unit, University College London, UK; 7 MRC Biostatistics Unit, Cambridge, UK

**Keywords:** Adherence, antidepressant, depression, primary care, treatment outcome

## Abstract

**Background:**

Meta-analyses suggest that reboxetine may be less effective than other antidepressants. Such comparisons may be biased by lower adherence to reboxetine and subsequent handling of missing outcome data. This study illustrates how to adjust for differential non-adherence and hence derive an unbiased estimate of the efficacy of reboxetine compared with citalopram in primary care patients with depression.

**Method:**

A structural mean modelling (SMM) approach was used to generate adherence-adjusted estimates of the efficacy of reboxetine compared with citalopram using GENetic and clinical Predictors Of treatment response in Depression (GENPOD) trial data. Intention-to-treat (ITT) analyses were performed to compare estimates of effectiveness with results from previous meta-analyses.

**Results:**

At 6 weeks, 92% of those randomized to citalopram were still taking their medication, compared with 72% of those randomized to reboxetine. In ITT analysis, there was only weak evidence that those on reboxetine had a slightly worse outcome than those on citalopram [adjusted difference in mean Beck Depression Inventory (BDI) scores: 1.19, 95% confidence interval (CI) –0.52 to 2.90, *p* = 0.17]. There was no evidence of a difference in efficacy when differential non-adherence was accounted for using the SMM approach for mean BDI (–0.29, 95% CI –3.04 to 2.46, *p* = 0.84) or the other mental health outcomes.

**Conclusions:**

There was no evidence of a difference in the efficacy of reboxetine and citalopram when these drugs are taken and tolerated by depressed patients. The SMM approach can be implemented in standard statistical software to adjust for differential non-adherence and generate unbiased estimates of treatment efficacy for comparisons of two (or more) active interventions.

## Introduction

Antidepressants are often prescribed in primary care as the first-line treatment for depression. In England in 2011, 46 million prescriptions for antidepressants were issued at a cost of £270 million (HSCIC, 2012). Selective serotonin reuptake inhibitors (SSRIs) are the most commonly prescribed (54% of prescriptions in 2011), with tricyclic antidepressants (TCAs) accounting for a further 29% of prescriptions issued (HSCIC, 2012).

Data on the comparative effectiveness of the various antidepressants suggest that there is little difference between different antidepressants (Freemantle *et al.* 2000; Cipriani *et al.* 2009). Two meta-analyses suggest that reboxetine may be less effective (Cipriani *et al.* 2009; Eyding *et al.* 2010) but others have reported no such differences (Papakostas *et al.* 2008).

Reboxetine is a selective noradrenaline reuptake inhibitor (NaRI), and is the only drug of this class of antidepressants currently licensed in the UK. It is prescribed infrequently (0.1% of total prescriptions for antidepressants in 2011) (HSCIC, 2012). Notably, meta-analyses have highlighted a lower adherence to treatment with reboxetine compared with other antidepressants (Cipriani *et al.* 2009; Eyding *et al.* 2010). This differential non-adherence poses problems when examining the results of randomized controlled trials (RCTs) comparing two active treatments because commonly used methods to handle missing data may lead to biased estimates of effectiveness. In the meta-analysis by Cipriani *et al.* (2009), it was assumed that those patients who were missing outcome data had not responded to treatment. However, as reboxetine was less well tolerated than SSRIs, this imputation has the potential to introduce bias such that the outcome for those on reboxetine may seem less favourable. Similarly, meta-analysis of trials that have used a last observation carried forward (LOCF) approach to handling missing outcome data (Eyding *et al.* 2010) may be biased in a similar direction. However, neither study explored the potential for bias based on their approach to dealing with missing data.

Importantly, these meta-analyses have focused on treatment effectiveness, that is the average outcome of the ‘offer’ of treatment obtained from intention-to-treat (ITT) analyses, irrespective of adherence to the allocated treatment. However, once it has been established that a medication can be tolerated by a patient, clinicians are often interested in knowing the benefit conferred by that drug when taken as prescribed. There is therefore clinical utility in estimating the efficacy of the drug under ‘ideal conditions’ (Last, 1995), which includes full adherence to treatment. Estimates of treatment efficacy from ‘per-protocol’ analyses may be biased (Fleming, 2008), and are further complicated in trials of two (or more) active interventions when there is differential adherence to the allocated treatments. A structural mean modelling (SMM) approach to deal with the issue of non-adherence in trials of two active treatments has been proposed by Fischer *et al.* (2011).

The current study had two aims. First, to test whether two commonly used approaches to dealing with missing data introduce bias in estimates of effectiveness derived in the presence of differential non-adherence between treatment arms. Second, to use data from the GENetic and clinical Predictors Of treatment response in Depression (GENPOD) trial (Lewis *et al.* 2011; Wiles *et al.* 2012) to illustrate how to adjust for differential non-adherence in an RCT of two active interventions and hence to derive an unbiased estimate of the efficacy of reboxetine compared with citalopram in the treatment of primary care patients with a new episode of depression.

## Method

### The GENPOD trial

The GENPOD trial (Thomas *et al.* 2008) was designed to test two primary hypotheses regarding (1) genetic and (2) clinical predictors of response to antidepressant medication. There was no evidence that the genetic serotonin polymorphism 5-HTTLPR (Lewis *et al.* 2011) or severity of depression (Wiles *et al.* 2012) was associated with response to antidepressant medication. Secondary analysis of these trial data can provide information on the comparative efficacy of an SSRI (citalopram) and an NaRI (reboxetine).

### Participants

Following agreement that an antidepressant should be prescribed, general practitioners (GPs) referred patients to the research team. Those eligible were aged 18–74 years, had a Beck Depression Inventory (BDI; Beck *et al.* 1996) score of ⩾15 and met ICD-10 criteria for a depressive episode (F32) using the computerized Clinical Interview Schedule – Revised (CIS-R; Lewis *et al.* 1992; Lewis, 1994). Those who gave written informed consent were randomized to receive either the SSRI citalopram (20 mg daily) or the NaRI reboxetine (4 mg twice daily).

Patients with psychosis, bipolar disorder or major substance or alcohol abuse problems were excluded, as were those who had taken antidepressants in the 2 weeks prior to baseline or who could not complete self-administered questionnaires.

### Baseline measures

In addition to age, gender, BDI score and CIS-R score, the following data were recorded at baseline: ethnicity, marital status, employment status, financial strain [based on questions from the Breadline Britain survey (Gordon *et al.* 2000) and a single question asking about how they were managing financially (five response options)], details of home ownership (home owner, tenant, other), whether they had any longstanding illness, disability or infirmity, total number of physical symptoms (based on a list of 28 symptoms), history of depression (self/family) and prior treatment for depression, personality – conscientiousness [Big Five Inventory (BFI); John *et al.* 1991], Hospital Anxiety and Depression Scale (HADS; Zigmond & Snaith, 1983) score, life events, social support, alcohol use (Alcohol Use Disorders Identification Test for Primary Care, AUDIT-PC; Piccinelli *et al.* 1997), and scores on the 12-item Short Form Health Survey (SF-12) mental and physical subscales (Jenkinson & Layte, 1997).

### Randomization procedure

Randomization was conducted by means of a computer-generated code, administered centrally and communicated by telephone and hence concealed from the recruiting researcher. Allocation was stratified by severity of overall symptoms (CIS-R score < 28 or ⩾28) and centre. The researcher gave the allocated medication to the participant. Neither patients nor researchers were blind to treatment allocation.

### Allocated treatments

Patients randomized to citalopram were prescribed 20 mg daily. Citalopram taken at this dose has been shown to occupy about 80% of serotonin transporter reuptake sites, which is reported to be the level of occupancy needed to produce reliable antidepressant effects (Meyer *et al.* 2001).

Those randomized to reboxetine were advised to start on 2 mg twice daily and increase to 4 mg twice daily after 4 days. This stepped approach to starting reboxetine treatment was used on the advice of psychopharmacologists to minimize problems with lack of tolerance of this drug. Acute doses of 4 mg of reboxetine increase cortisol levels indicative of increased noradrenergic function (Hill *et al.* 2003) and this dose of drug also produces peripheral autonomic effects consistent with noradrenaline reuptake blockade (Szabadi *et al.* 1998). GPs could increase the dose of either allocated treatment if deemed clinically appropriate.

### Measures of treatment adherence

Participants were asked about their use of antidepressant medication in the follow-up questionnaires (six closed response options: I have not taken any of my tablets; I have taken hardly any of my tablets; I have taken less than half of my tablets; I have taken more than half of my tablets; I have taken nearly all my tablets; I have taken my tablets every day).

### Outcome measures

Self-reported outcome data were collected 6 and 12 weeks after randomization. For the purpose of this study, which demonstrates the approach to adjusting for differential non-adherence between the two treatments, we used the 6-week outcome data. The (original) primary outcome was the total BDI score at 6 weeks. Secondary outcomes were the HADS total and subscale scores and the SF-12 mental and physical subscale scores.

### Dataset

The 6-week follow-up was completed by 91% of participants (*n* = 546) [citalopram: 274/298 (92%) and reboxetine: 272/303 (90%)]. Younger individuals, those with more life events and less social support were more likely to have missing data (Lewis *et al.* 2011). Adjustment for these variables made no difference to the main trial findings (Lewis *et al.* 2011) and there was no evidence that these factors were associated with adherence to medication (data not shown). Therefore, for the present analyses, the dataset comprised the 546 participants with 6-week follow-up data (complete cases).

### Statistical analysis

All analyses were conducted in Stata version 11.1 (Stata Corporation, USA). To compare the data from the GENPOD trial with the previous literature on the comparative effectiveness of antidepressants (Papakostas *et al.* 2008; Cipriani *et al.* 2009; Eyding *et al.* 2010), we first conducted analyses on the effectiveness of reboxetine *versus* citalopram according to the ITT principle. We then examined the effect of two approaches to handling missing data that have been used in the previous meta-analyses to illustrate the potential for bias in such estimates of effectiveness in the presence of differential non-adherence. Finally, we focused on the application of the novel SMM approach to estimating treatment efficacy in the presence of differential non-adherence.

### Estimates of effectiveness

The primary comparative ITT analysis compared the BDI score at 6 weeks between the two groups as randomized, with adjustment for baseline BDI score and the stratification variables. To estimate treatment effectiveness, data from all participants followed up at 6 weeks were included in these analyses, irrespective of adherence to the allocated medication.

### Effect of imputing missing outcomes as ‘non-recovery’ or using an LOCF approach to handling missing outcome data on estimates of effectiveness

Previous studies comparing outcomes for those taking citalopram and reboxetine (Cipriani *et al.* 2009; Eyding *et al.* 2010) analysed data on an ITT basis but either: (1) assumed that those who were missing outcome data (which frequently equates to all those who had stopped the trial medication in psychopharmacology trials) had not responded to treatment (Cipriani *et al.* 2009) or (2) summarized data from publications that used an LOCF approach to handle missing data (Eyding *et al.* 2010). The effect of these two different approaches to handling missing data was examined by artificially constraining the GENPOD dataset such that only those who had continued to take their medication at 6 weeks were regarded as having outcome data.

### Adherence-adjusted efficacy estimates

The final set of analyses generated unbiased estimates of treatment efficacy in the presence of differential non-adherence between treatment arms. The SMM method assumes that the mean outcomes in the two arms would be equal in the absence of treatment, and that each treatment has a (separate) linear causal effect on outcome. To estimate the two causal effects of treatment, the approach developed by Fischer *et al.* (2011) relies on identifying baseline variables that predict adherence differently in the two arms (i.e. they interact with a randomized group in a model for adherence) but that do not predict the causal effect of treatment (i.e. they do not interact with treatment in a causal model for clinical outcome). Baseline variables that predict adherence and/or outcome (as main effects) are also useful in improving precision. The following procedure was used to identify these baseline variables.

#### (1) Identifying predictors of outcome

All baseline variables that were possible predictors of outcome [age, gender, ethnicity, marital status, employment status, housing status, financial strain, history of depression (self/family), prior treatment for depression, longstanding illness, disability/infirmity, social support, life events, alcohol score, BDI score, HADS total/anxiety/depression subscale scores, SF-12 mental and physical subscale scores, and number of physical symptoms] were examined in univariable linear regression models with the BDI score at 6 weeks as a continuous outcome. Those variables that were identified as predictors of outcome at *p* < 0.20 were entered into a multivariable model. The most parsimonious model was identified using backwards selection and the likelihood ratio test until all remaining variables were retained at *p* < 0.10. Any variables not selected in the initial phase (univariable model: *p* ⩾0.20) were included in the final multivariable model one by one and retained if *p* < 0.10. This modelling process was repeated for each of the additional outcomes (HADS total and subscale scores and SF-12 mental and physical subscale scores). All models were adjusted for stratification variables and treatment allocation to improve precision.

This liberal modelling approach ensured that all potentially influential variables were included. Omission of a potentially important predictor of outcome from the SMM model would result in a loss of precision.

#### (2) Identifying predictors of adherence

GENPOD relied upon self-reported use of antidepressant medication. A quantitative measure of adherence is required for the SMM approach. Therefore, a pragmatic decision was made to rescale the six response options using increments of 0.2 to generate an adherence score scaled from zero to one, where zero represented total non-adherence and one indicated ‘perfect’ adherence. This rescaling of the adherence measure assumed that a 0.2 point increase in adherence had the same meaning across the scale.

The following baseline variables were possible predictors of adherence: sociodemographic factors (age, gender, ethnicity, marital status, employment status, housing status, financial strain), social support, history of depression (self/family)/prior treatment for depression, longstanding illness/disability/infirmity, personality – conscientiousness, life events, alcohol use, SF-12 physical subscale score, and eight physical symptoms (rapid heartbeat, agitation, dry mouth, sweating, constipation, diarrhoea, daytime drowsiness, and hot flushes). The total number of physical symptoms at baseline was excluded from the list because it was thought that individual physical symptoms may be more relevant to the question of adherence. For example, if someone was already experiencing a dry mouth, taking a drug likely to affect this may differentially affect adherence. The possible predictors of adherence were initially examined in univariable linear regression models with adherence score as the outcome, with adjustment for treatment allocation and predictors of outcome (identified using the process described earlier). All variables that were identified as predictors of adherence (either as a main effect or an interaction with treatment allocation in the univariable models at *p* < 0.20) were entered into a multivariable model with the variable specified in the appropriate form (main effect or main effect and interaction). Interactions were evaluated one at a time using the likelihood ratio test. Those variables for which the main effect or interaction was significant at *p* < 0.10 were retained in the final multivariable model.

In GENPOD, the primary hypotheses were about differential response to antidepressant treatment dependent on severity of depression and genotype. To be consistent with this hypothesis, it was deemed inappropriate to examine severity as a predictor of adherence to medication because severity may have predicted the effect of treatment other than through adherence. Therefore, all measures of severity of depressive symptoms (CIS-R, BDI, HADS and SF-12 mental subscale score) were excluded from the list of potential predictors of adherence.

#### (3) Generating adherence-adjusted estimates

The SMM approach (Fischer *et al.* 2011) was implemented using an instrumental variable (IV) model approach in Stata [ivregress command: two-stage least-squares (2sls) approach] for each of the outcomes (BDI, HADS and SF-12 mental and physical subscale scores). Each model was specified in the following format:
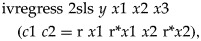
where *y* = outcome, *x*1 = list of predictors of outcome (identified in stage 1), *x*2 = list of predictors of adherence (identified in stage 2), *x*3 = stratification variables (centre and CIS-R severity stratum), c1 = adherence score for those randomized to treatment group 1 (citalopram), c2 = adherence score for those randomized to treatment group 2 (reboxetine), r = treatment allocation, and * denotes an interaction, e.g. r*x1= interaction between treatment allocation and predictors of outcome.

The SMM method requires identification of baseline variables that predict adherence differentially in the two arms (Fischer *et al.* 2011). These variables were included in *x*2 and not in *x*1, so the interaction r*x2 was an essential part of the model specification whereas the interaction r*x1 is unlikely to be important and could be omitted. Variables that may modify the causal effect of treatment should not be included in *x*1 or *x*2.

Taking outcome as BDI score at 6 weeks as an example, the IV model estimated the causal effects of full adherence to the two treatments (citalopram and reboxetine); that is, the difference in mean BDI scores for full adherence with the treatment compared to no adherence with any treatment. The difference between the two treatments was then tested formally using the lincom command (lincom c2 – c1), which estimates an adherence-adjusted difference in mean BDI scores between the two treatment groups and its 95% confidence interval (CI).

Sensitivity analyses were conducted removing predictors of adherence from the list of *x*2 variables one by one to examine the robustness of the findings from the SMM IV approach for each of the outcomes.

## Results

### Trial participation and follow-up

The Consolidated Standards Of Reporting Trials (CONSORT) flowchart and baseline comparability of the randomized groups have been published previously (Lewis *et al.* 2011). In total, 601 participants were randomized to receive either citalopram (*n* = 298) or reboxetine (*n* = 303). The mean age of participants was 38.8 years (s.d. = 12.4) and 68% (*n* = 408) were female. More than 90% of participants had moderate (*n* = 305) or severe depression (*n* = 245) according to ICD-10 criteria. The 6-week follow-up was completed by 91% (*n* = 546) of participants (citalopram: *n* = 274 and reboxetine: *n* = 272).

### Adherence to, and dose of, medication

Of those randomized to citalopram, 90% (*n* = 246) were still taking their medication at the time of the 6-week follow-up, compared with 72% (*n* = 195) of those randomized to reboxetine (difference: 18.4%, 95% CI 12.0–24.8, *p* < 0.001). At the 6-week follow-up, 149 (55%) of those randomized to receive citalopram reported having taken their tablets ‘every day’, 90 (33%) had taken ‘nearly all’ their tablets, and 34 (12%) had taken ‘less than half’, ‘hardly any’ or none of their tablets. The comparable figures for those randomized to receive reboxetine were 113 (42%), 89 (33%) and 70 (26%). As reported previously (Lewis *et al.* 2011), the dose of the allocated medication was increased by the GP only for a minority of participants [citalopram: *n* = 55 (20%); reboxetine: *n* = 13 (5%)] during the trial.

### Estimates of effectiveness

Among the 546 participants who completed the 6-week follow-up, their mean BDI score at baseline was 33.6 (s.d. = 9.7). The corresponding figures by trial arm are given in Table 1. In an ITT analysis (Table 2), there was only weak evidence to suggest that those randomized to reboxetine had a worse outcome. On average, those on reboxetine scored one point higher on the BDI, although the 95% CI included no difference between groups. The results of the effectiveness analyses for the other mental health outcome measures (HADS total and anxiety/depression subscales; SF-12 mental subscale) were consistent with this (Table 2). Hence, those randomized to receive reboxetine had, on average, a higher score on the HADS (total and subscales) and a lower score on the SF-12 mental subscale, indicative of a worse outcome. Indeed, for the SF-12 mental health subscale, those randomized to reboxetine had a mean score that was, on average, two points lower compared to those randomized to receive citalopram. The CI surrounding this estimate excluded the possibility of no difference. There was little evidence for any difference in outcome in terms of physical health (SF-12 physical subscale score) between those randomized to receive reboxetine compared to citalopram (Table 2).

**Table 1.** Baseline and 6-week follow-up scores on the outcome measures according to allocated treatment group, in those who completed the 6-week follow-up

Outcome	Time point	Citalopram (*n* = 274)^a^		Reboxetine (*n* = 272)	
		Mean	s.d.	Mean	s.d.
BDI	Baseline	34.2	9.3	33.1	10.0
	6 weeks	18.9	10.8	19.6	11.1
HADS Total	Baseline	26.1	6.0	25.5	6.4
	6 weeks	17.2	8.3	18.0	8.4
HADS Anxiety	Baseline	13.3	3.4	13.1	3.8
	6 weeks	9.3	4.2	9.7	4.4
HADS Depression	Baseline	12.8	3.8	12.4	4.1
	6 weeks	7.9	4.9	8.3	5.0
SF-12 Mental	Baseline	23.9	7.6	24.3	7.8
	6 weeks	39.2	11.9	37.2	12.4
SF-12 Physical	Baseline	47.2	11.5	47.4	11.1
	6 weeks	48.2	10.6	47.8	10.2

BDI, Beck Depression Inventory; HADS, Hospital Anxiety and Depression Scale; SF-12, 12-item Short-Form Health Survey; s.d., standard deviation.

a *n* = 273 for SF-12 scores.

**Table 2.** Differences in outcomes at 6 weeks from analysis of treatment effectiveness and estimates of efficacy from SMM models that account for differential non-adherence to allocated treatment

	Estimates of effectiveness from analyses conducted according to the ITT principle				Adherence-adjusted efficacy estimates			
Outcome	*n*	Adjusted difference in means^a^	95% CI	*p* value	*n*	Adjusted difference in means^a^	95% CI	*p* value
BDI	546	1.19	−0.52 to 2.90	0.17	541	−0.29	−3.04 to 2.46	0.84
HADS Total	546	1.16	−0.16 to 2.47	0.09	543	1.23	−0.71 to 3.17	0.22
HADS Anxiety	546	0.56	−0.09 to 1.21	0.09	543	0.57	−0.43 to 1.57	0.27
HADS Depression	546	0.60	−0.17 to 1.37	0.13	543	0.62	−0.50 to 1.74	0.28
SF-12 Mental	545	−2.22	−4.21 to –0.22	0.03	543	−2.53	−5.55 to 0.50	0.10
SF-12 Physical	545	−0.54	−1.86 to 0.77	0.42	543	−0.61	−2.52 to 1.31	0.53

ITT, Intention-to-treat; BDI, Beck Depression Inventory; HADS, Hospital Anxiety and Depression Scale; SF-12, 12-item Short-Form Health Survey; CI, confidence interval.

a Adjusted for centre, baseline severity strata (Clinical Interview Schedule – Revised, CIS-R) and baseline score for outcome measure.

Difference is reboxetine minus citalopram. A positive difference for BDI and HADS (and a negative difference for SF-12 outcomes) indicates that those on reboxetine have a worse outcome than those on citalopram.

### Effect of imputing missing outcomes as ‘non-recovery’ or using an LOCF approach to handling missing outcome data on estimates of effectiveness

There was little evidence of a difference in the binary outcome of ‘recovery’ (BDI score < 10 at 6 weeks) using observed data collected (irrespective of adherence to allocated medication) for 91% of GENPOD participants at 6 weeks when data were analysed using an ITT approach (Table 3).

**Table 3.** Examining the effect of different approaches to handling missing outcome data on the difference between treatment groups (estimates of effectiveness) in the presence of differential adherence to treatment

Outcome	Analysis	Citalopram		Reboxetine		Difference between groups OR (95% CI)^a^	*p* value
		*N*	*n* (%)	*N*	*n* (%)		
‘Recovery’ (BDI score < 10)	ITT on observed data (*n* = 546)	274	60 (21.9)	272	61 (22.4)	0.95 (0.62 to 1.44)	0.79
	Using observed outcome data for those who were continuing to take their allocated medication at 6 weeks and assuming that those who had stopped their allocated medication had not recovered (*n* = 546)	274	55 (20.1)	272	44 (16.2)	0.70 (0.45 to 1.10)	0.12
	As above and assuming those with missing outcome data were also non-responders (*n* = 601)	298	55 (18.5)	303	44.9 (14.5)	0.70 (0.45 to 1.09)	0.11
		*N*	Mean (s.d.)	*N*	Mean (s.d.)	Difference in means (95% CI)^a^	
BDI score	ITT on observed data (*n* = 546)	274	18.9 (10.8)	272	19.6 (11.1)	1.19 (−0.52 to 2.90)	0.17
	Using observed outcome data for those who were continuing to take their allocated medication at 6 weeks and using an LOCF approach to carry forward the baseline BDI score for those who had stopped their allocated medication (*n* = 546)	274	20.2 (11.8)	272	22.6 (12.0)	3.01 (1.18 to 4.85)	0.001

BDI, Beck Depression Inventory; ITT, intention-to-treat; LOCF, last observation carried forward; OR, odds ratio; CI, confidence interval; s.d., standard deviation.

a Adjusted for centre, baseline severity strata (Clinical Interview Schedule – Revised, CIS-R) and baseline BDI score.

Difference is reboxetine minus citalopram. An OR < 1 for ‘recovery’ or a positive difference for differences in BDI scores indicates that those on reboxetine have a worse outcome compared to those on citalopram.

Applying the assumption that those who stopped their medication had a poor outcome to the GENPOD data demonstrated that differential adherence to medication between arms introduced bias such that the outcome for those randomized to reboxetine appeared worse [odds ratio (OR) for response 0.70, 95% CI 0.45–1.10)]. Additional imputation of a poor outcome for those individuals not followed up at 6 weeks had little effect (Table 3).

Similarly, using an LOCF approach to impute missing outcome data for those who had stopped their medication at 6 weeks suggested that, on average, the outcome for those randomized to reboxetine was three points higher on the BDI (more depressed) compared with those randomized to citalopram. Analysis of the observed outcome data at 6 weeks provided only weak evidence for a difference in outcome between the groups (Table 3).

### Adherence-adjusted efficacy estimates

The analyses identified several predictors of outcome and adherence within the GENPOD dataset (see the online Appendix). As expected, for all outcomes, the strongest predictor of outcome was the baseline measurement. In terms of predictors of adherence, those from a non-white ethnic background were less likely to adhere to medication, whereas those who reported a rapid heartbeat were more likely to adhere to medication. Interactions with treatment allocation were found for three variables: marital status, prior history of depression and the personality trait of conscientiousness. Those who were married, those with a previous history of depression and those who were more conscientious were less likely to adhere to reboxetine. The full specification of the IV models that generated the adherence-adjusted estimates can be found in the online Appendix.

The adherence-adjusted differences in mean outcomes between the treatment groups are presented in Table 2. There was weak evidence that reboxetine was less efficacious than citalopram in terms of outcome on the SF-12 mental subscale, although the CI included the possibility of no difference. However, there was no evidence of a difference in efficacy between the two treatments based on the other outcomes including the BDI.

### Sensitivity analyses for the adherence-adjusted efficacy estimates

The results of the sensitivity analyses examining the effect of removing predictors of adherence from the final SMM IV models for all outcomes are summarized in Table 4. Although the adjusted difference in means between treatment groups varied according to the list of predictors of adherence included in the SMM model (for some outcomes more than others), the estimates were broadly consistent when the CIs were compared.

**Table 4.** Sensitivity analyses around adherence-adjusted instrumental variable (IV) efficacy estimates of the mean difference in outcome between treatment groups

Outcome 〈Y〉	Method	*n*	Adjusted difference^a^ in mean 〈Y〉 score at 6 weeks	95% CI	*p* value
BDI	‘Adherence-adjusted’ efficacy estimate	541	−0.29	−3.04 to 2.46	0.84
	**Excluding predictors of adherence from list of *x*2's one by one**				
	Ethnicity	541	−0.24	−3.03 to 2.54	0.87
	Rapid heart beat	541	−0.17	−2.94 to 2.60	0.90
	Marital status	541	−0.77	−3.88 to 2.33	0.62
	History of depression	541	−0.46	−3.34 to 2.42	0.76
	BFI conscientiousness score	541	0.29	−2.59 to 3.16	0.84
HADS Total	‘Adherence-adjusted’ efficacy estimate	543	1.23	−0.71 to 3.17	0.22
	**Excluding predictors of adherence from list of *x*2's one by one**				
	Ethnicity	543	1.34	−0.64 to 3.31	0.18
	Rapid heart beat	543	1.13	−0.82 to 3.08	0.26
	Marital status	543	0.94	−1.17 to 3.04	0.38
	History of depression	543	1.32	−0.67 to 3.32	0.19
	BFI conscientiousness score	543	1.29	−0.76 to 3.34	0.22
HADS Anxiety	‘Adherence-adjusted’ efficacy estimate	543	0.57	−0.43 to 1.57	0.27
	**Excluding predictors of adherence from list of *x*2's one by one**				
	Ethnicity	543	0.66	−0.35 to 1.68	0.20
	Rapid heart beat	543	0.52	−0.48 to 1.52	0.31
	Marital status	543	0.49	−0.58 to 1.56	0.37
	History of depression	543	0.57	−0.47 to 1.60	0.28
	BFI conscientiousness score	543	0.55	−0.50 to 1.60	0.31
HADS Depression	‘Adherence-adjusted’ efficacy estimate	543	0.62	−0.50 to 1.74	0.28
	**Excluding predictors of adherence from list of *x*2's one by one**				
	Ethnicity	543	0.63	−0.50 to 1.76	0.28
	Rapid heart beat	543	0.56	−0.57 to 1.69	0.33
	Marital status	543	0.43	−0.77 to 1.63	0.48
	History of depression	543	0.72	−0.44 to 1.87	0.22
	BFI conscientiousness score	543	0.64	−0.54 to 1.83	0.29
SF-12 Mental	‘Adherence-adjusted’ efficacy estimate	543	−2.53	−5.55 to 0.50	0.10
	**Excluding predictors of adherence from list of *x*2's one by one**				
	Ethnicity	543	−2.80	−5.89 to 0.29	0.08
	Rapid heart beat	543	−2.29	−5.32 to 0.75	0.14
	Marital status	543	−2.85	−6.11 to 0.41	0.09
	History of depression	543	−2.97	−6.10 to 0.17	0.06
	BFI conscientiousness score	543	−2.01	−5.21 to 1.19	0.22
SF-12 Physical	‘Adherence-adjusted’ efficacy estimate	543	−0.61	−2.52 to 1.31	0.53
	**Excluding predictors of adherence from list of *x*2's one by one**				
	Ethnicity	543	−0.83	−2.80 to 1.13	0.41
	Rapid heart beat	543	−0.65	−2.57 to 1.27	0.51
	Marital status	543	0.13	−1.95 to 2.21	0.90
	History of depression	543	−0.96	−2.97 to 1.05	0.35
	BFI conscientiousness score	543	−0.82	−2.84 to 1.20	0.43

BDI, Beck Depression Inventory; HADS, Hospital Anxiety and Depression Scale; SF-12, 12-item Short-Form Health Survey; BFI, Big Five Inventory; CI, confidence interval.

a Adjusted for centre, baseline severity strata (Clinical Interview Schedule – Revised, CIS-R) and baseline score for outcome measure.

There was no evidence to support an interaction between severity of depression (or genotype) and response to antidepressant in the GENPOD trial (Lewis *et al.* 2011; Wiles *et al.* 2012). Therefore, excluding severity as a predictor of adherence may be questioned. However, there was no evidence for an interaction between severity of depression and adherence to medication (for test of equality of coefficients: interaction between severity and adherence to citalopram/reboxetine, *p* = 0.27).

## Discussion

We have demonstrated how to implement the SMM approach described by Fischer *et al*. (2011) in a standard statistical software package to obtain an unbiased estimate of treatment efficacy for a trial comparing two active treatments. Analysis was straightforward once suitable covariates for the SMM approach were identified. Data from the GENPOD trial of the two antidepressants citalopram and reboxetine were used as an exemplar.

The results of an effectiveness analysis (conducted according to the ITT principle) found only weak evidence that those randomized to reboxetine had a slightly worse outcome than those randomized to citalopram in terms of depressive symptoms (on the BDI/HADS). This is in contrast to previous meta-analyses (Cipriani *et al.* 2009; Eyding *et al.* 2010) that suggested that reboxetine was less effective than other antidepressants.

It is common practice in psychopharmacology trials for participants who stop taking their allocated medication not to be followed up. Outcomes are then imputed by assuming that those who stopped their allocated medication had a poor outcome or by carrying forward an earlier observation (LOCF). When we applied these approaches to the GENPOD data, by artificially assuming that outcomes were observed only for those who continued on their medication, we found stronger evidence of a poor outcome for those randomized to reboxetine compared with the results of analyses using all observed data. This clearly demonstrates that these common approaches to handling missing data may generate biased estimates of effectiveness when there is differential non-adherence between treatment arms.

Using the SMM approach to account for differential non-adherence to treatment between trial arms, we found no evidence of a difference in efficacy in terms of depressive symptoms (BDI) between reboxetine and citalopram at 6 weeks. The adherence-adjusted estimate (based on the difference in causal effects for full adherence to the treatment) was close to the null. There was weak evidence for a difference in efficacy between treatment with reboxetine and citalopram for the SF-12 mental subscale. In discussing these differences, it is important to consider whether these are clinically relevant. Although there is no consensus regarding a ‘minimum clinically important difference’ on these outcome scales, a change of 0.33 s.d. is often used as the target difference in primary care depression trials (Baxter *et al.* 2010). Hence, we would regard a three-point change in BDI score, a two-point change in HADS score (one point on subscales) and a three- to four-point change in SF12 scores to be clinically important. The differences and CIs observed in terms of estimates of efficacy from analyses using the SMM approach are smaller than these and, except for the results for the SF-12 mental subscale, we can therefore exclude the possibility of a clinically important difference between citalopram and reboxetine in those who can tolerate the medications.

### Strengths and limitations

The SMM approach used depends on finding baseline covariates that predict adherence differently in the two randomized groups but that may be assumed not to modify the causal effect of treatment. Bias would occur if the latter assumption failed. In addition, it is assumed that the average outcome does not depend on treatment assignment (the ‘exclusion restriction’). In a non-blinded trial such as GENPOD, there is a theoretical possibility that this assumption could be violated given prior beliefs about the treatment. However, there is little evidence to suggest that patients had different expectations of outcome for the two antidepressants.

Predictors of adherence were removed from the final SMM IV models one at a time to examine the robustness of the findings. The results of these sensitivity analyses show that the estimates were broadly consistent with the final SMM model incorporating all predictors of adherence.

GENPOD relied upon a self-report measure of adherence to medication. Use of electronic monitoring bottles would provide a more accurate measure of adherence. Such data would also provide a continuous adherence score as required for application of the SMM methodology. We rescaled the self-report adherence data to generate a continuous measure of adherence to apply this methodology, albeit therefore introducing some modelling assumptions. At the same time, there was no reason for participants to be motivated to mislead the researchers about their use of medication and we therefore have no reason to suppose that this measure was biased.

In total, 601 participants were recruited into the GENPOD trial, making this one of the largest primary care depression trials conducted. Nonetheless, despite its large size, it is of note that estimates obtained from models based on instrumental variables methods remain imprecise.

### Comparisons with existing literature for comparative effectiveness of antidepressants

Meta-analyses have suggested that reboxetine may be less effective than other antidepressants (Cipriani *et al.* 2009; Eyding *et al.* 2010). However, in effectiveness analyses of data from the GENPOD trial, we found only weak evidence of very small differences in mental health outcomes (that were unlikely to be clinically significant) at 6 weeks for those randomized to reboxetine compared with citalopram. Both meta-analyses (Cipriani *et al.* 2009; Eyding *et al.* 2010) reported that patients randomized to reboxetine were more likely to discontinue treatment compared with those randomized to SSRIs, which is consistent with the findings from GENPOD. However, as we have demonstrated, the assumption that individuals with missing outcome data have not responded to treatment may introduce bias in estimates of effectiveness, such that those on reboxetine seem to do worse. It is therefore important to continue to follow-up trial participants to collect outcome data even if they stop taking the trial medication.

### Extensions to the SMM methodology

We have described the SMM approach for estimating efficacy for a singly-measured quantitative outcome. For a repeated-measured quantitative outcome, a structural nested mean model could be used (Robins, 1994). For a binary outcome, the SMM approach can be used to estimate risk differences, but if interest lies in risk ratios or ORs then a multiplicative SMM or a generalized SMM is needed (Vansteelandt & Goetghebeur, 2003). For time-to-event outcomes, rank-preserving structural nested failure time models could be used (Robins & Tsiatis, 1991).

The methods we have described are especially appropriate for equivalence and non-inferiority trials because ITT analysis is known to be anti-conservative in such trials (Jones *et al.* 1996) whereas per-protocol analyses are potentially biased (Fleming, 2008). An alternative approach to handling non-adherence is the complier average causal effect (CACE; Dunn *et al.* 2003) model, but this is not well defined in trials comparing two active treatments and also requires adherence to be binary. Dichotomizing a continuous adherence measure is usually undesirable (White *et al.* 2011).

### Implications and further research

It is common practice in RCTs of pharmacological interventions for participants not to be followed up if they stop taking the trial medication. Such a policy is at odds with conducting primary trial analyses according to the principle of ITT, and assumptions that are then made regarding missing data frequently bias estimates of effectiveness.

Differential non-adherence between treatment arms presents a particular challenge for trialists. However, as illustrated, it is possible to implement the analytical methods described (Fischer *et al.* 2011) in a standard statistical software package to take account of non-adherence to treatment when comparing two (or more) active interventions. Such methods will generate an unbiased estimate of the difference in treatment efficacy that is of value to the clinician in terms of describing the likely outcomes when drugs are both taken, and tolerated, by patients.

## Supplementary Material

Supplementary Material Supplementary information supplied by authors.

## Supplementary material

For supplementary material accompanying this paper visit http://dx.doi.org/10.1017/S0033291714000221.
