# Supplementary material for: Allowing for non-adherence to treatment in a randomized controlled trial of two antidepressants (citalopram versus reboxetine): an example from the GENPOD trial
Source: Psychol Med. 2014 Mar 3;44(13):2855–66. doi: 10.1017/S0033291714000221 (PMC4131263; doi:10.1017/S0033291714000221)
Supplement: Supplementary Material — Supplementary information supplied by authors. [file S0033291714000221sup001.doc]

**ONLINE APPENDIX**

**Table S1 – Predictors of outcome identified in GENPOD dataset in multivariable model at p<0.10**

| **Outcome at 6 weeks** | **Predictor** | **Coefficient** | **95%CI** | **p value** |
| --- | --- | --- | --- | --- |
| BDI | Baseline BDI score | 0.40 | 0.29, 0.51 | <0.001 |
|  | Age | 0.09 | 0.02, 0.16 | 0.015 |
|  | Social support score | -0.31 | -0.55, -0.07 | 0.010 |
|  | AUDIT (alcohol) score | -0.27 | -0.51, -0.03 | 0.025 |
|  | Total number of physical symptoms | 0.20 | 0.002, 0.39 | 0.048 |
| HADS (total) | Baseline HADS score | 0.42 | 0.30, 0.53 | <0.001 |
|  | Age | 0.09 | 0.03, 0.15 | 0.002 |
|  | Social support score | -0.19 | -0.37, -0.01 | 0.040 |
|  | Employment status | (7 categories for brevity report p only) | | 0.005 |
| HADS  (anxiety subscale) | Baseline HADs anxiety | 0.44 | 0.34, 0.55 | <0.001 |
|  | Employment status | (7 categories for brevity report p only) | | 0.002 |
| HADS (depression subscale) | Baseline HADS depression score | 0.43 | 0.33, 0.53 | <0.001 |
|  | Age | 0.06 | 0.02, 0.09 | 0.002 |
|  | Social support score | -0.14 | -0.25, -0.04 | 0.007 |
|  | Employment status | (7 categories for brevity report p only) | | 0.087 |
|  | AUDIT (alcohol) score | -0.11 | -0.22, -0.01 | 0.034 |
|  | Longstanding illness/disability | 0.76 | -0.04, 1.57 | 0.063 |
| SF-12 mental subscale | Baseline SF-12 mental subscale score | 0.32 | 0.17, 0.46 | <0.001 |
|  | Baseline BDI score | -0.16 | -0.29, -0.04 | 0.011 |
|  | Employment status | (7 categories for brevity report p only) | | 0.086 |
| SF-12 physical subscale | Baseline SF-12 physical subscale score | 0.55 | 0.48, 0.62 | <0.001 |
|  | Age | -0.11 | -0.17, -0.05 | 0.001 |
|  | AUDIT (alcohol) score, | 0.21 | 0.03, 0.39 | 0.019 |
|  | Employment status | (7 categories for brevity report p only) | | <0.001 |
|  | Baseline SF-12 mental subscale score | 0.09 | -0.003, 0.18 | 0.058 |

**All models adjusted for treatment allocation and stratification variables (centre and CISR stratum) n = 541*

**Table S2 – Predictors of adherence identified in GENPOD dataset in multivariable model at p<0.10 (for main effect or interaction) adjusted for predictors of BDI outcome at 6 weeks**

| **Predictors of adherence - main effect**  **or interaction with treatment allocation** | **Coefficient*** | **95%CI** | **p value†** |
| --- | --- | --- | --- |
| Non-white ethnic background | -0.10 | -0.21, 0.005 | 0.062 |
| Reporting symptoms of a rapid heartbeat | 0.05 | 0.003, 0.09 | 0.035 |
|  |  |  |  |
| *Marital status* |  |  | 0.066 |
| Married/living as married | 0 |  |  |
| Single | -0.05 | -0.13, 0.02 |  |
| Separated/Divorced/Widowed | -0.06 | -0.14, 0.03 |  |
| *Interaction:* Treatment (reboxetine) by Single | 0.11 | 0.005, 0.21 |  |
| Treatment (reboxetine) by S/D/W | 0.09 | -0.02, 0.21 |  |
|  |  |  |  |
| *History of depression* |  |  | 0.047 |
| Prior history of depression | 0.02 | -0.05, 0.09 |  |
| *Interaction:* Treatment (reboxetine) by history of depression | -0.10 | -0.20, 0.0001 |  |
|  |  |  |  |
| *Personality - Conscientiousness* |  |  | 0.018 |
| Conscientiousness (BFI) score | 0.03 | -0.01, 0.07 |  |
| *Interaction:* Treatment (reboxetine) by conscientiousness score | -0.07 | -0.13, -0.01 |  |

**Model (n=543) adjusted for treatment allocation and predictors of BDI outcome at 6 weeks (age, social support score, AUDIT (alcohol) score). Baseline BDI score was not included in the model as it was hypothesised (in the main trial) that this might modify the causal effect of treatment.*

***†****p value for main effect or interaction*

*Note: This is an exemplar – coefficients adjusting for predictors of other outcomes (not BDI) will be slightly different*

**Specification of IV models to generate adherence-adjusted estimates for each outcome**

Model as presented in main paper:

*ivregress 2sls* y x1 x2 x3 ( c1 c2 = r x1 r*x1 x2 r*x2)

where:

| y = outcome | c1 = adherence score for those randomised to treatment group 1 (citalopram) |
| --- | --- |
| x1 = list of predictors of outcome | c2 = adherence score for those randomised to treatment group 2 (reboxetine) |
| x2 = list of predictors of adherence | r = treatment allocation |
| x3 = stratification variables | * denotes an interaction e.g. r*x1 = interaction between treatment allocation and predictors of outcome |

**Table S**3 – List of variables included in the final instrumental variable regression models

| **Outcome at**  **6 weeks** | **Predictors of outcome (x1)** | **Predictors of adherence (x2)*** | **Stratification variables (x3)*** |
| --- | --- | --- | --- |
| BDI | - baseline BDI score - age - social support score - AUDIT (alcohol) score - number of physical symptoms | - ethnicity - rapid heartbeat - marital status - prior history of depression - conscientiousness score | - centre - CIS-R stratum |
| HADS  (total) | - baseline HADS score - age - employment status - social support score |
| HADS  (anxiety subscale) | - baseline HADS anxiety subscale score - employment status |
| HADS (depression subscale) | - baseline HADS depression subscale score - age - employment status - social support score - AUDIT (alcohol) score - longstanding illness/disability |
| SF-12 mental subscale | - baseline SF-12 mental subscale score - employment status - baseline BDI score |
| SF-12 physical subscale | - baseline SF-12 physical subscale score - age - employment status - AUDIT (alcohol) score - baseline SF-12 mental subscale score |

**variables included under list of predictors of adherence and stratification variables are the same for all outcomes listed; t0 = baseline measure of the outcome*
